# Supplementary figures and images for: The Toxoplasma monocarboxylate transporters are involved in the metabolism within the apicoplast and are linked to parasite survival
Source: eLife. 2024 Mar 19;12:RP88866. doi: 10.7554/eLife.88866 (PMC10950331; doi:10.7554/eLife.88866)

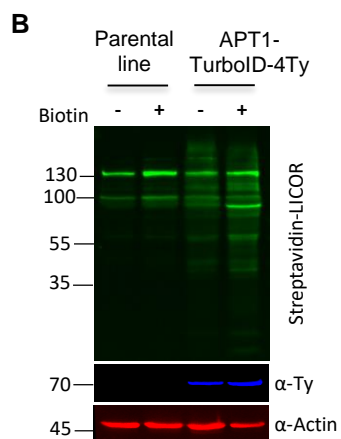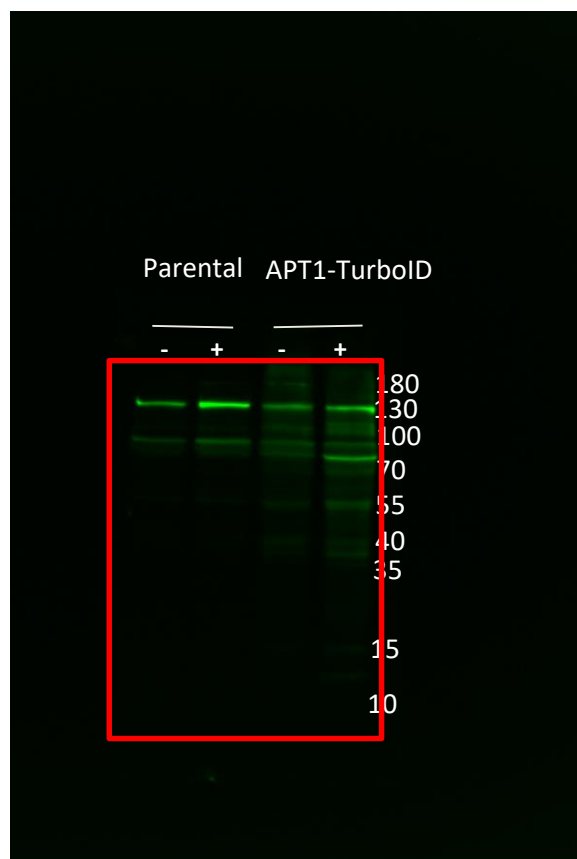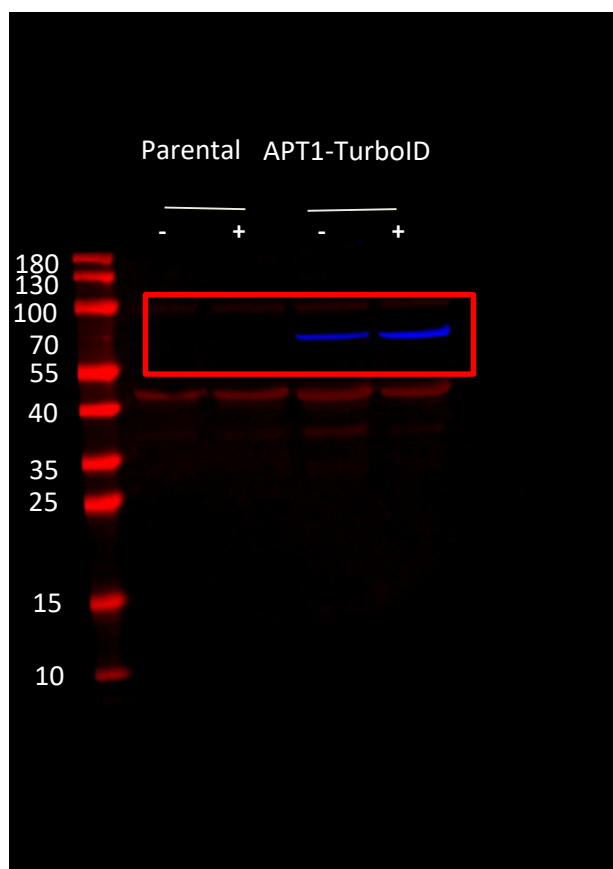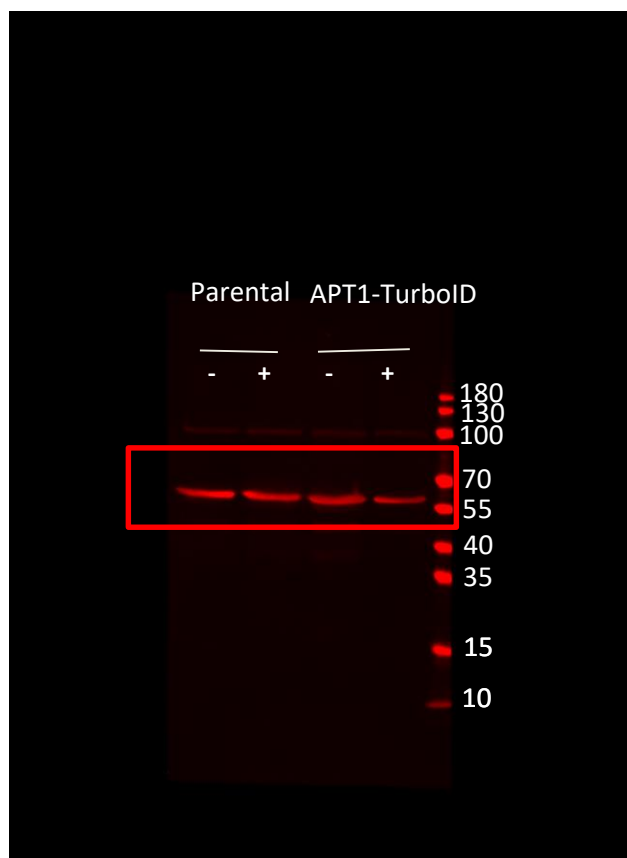

Supplement: Figure 1—source data 1. [file elife-88866-fig1-data1.zip › Figure 1-Source data 1.pdf]

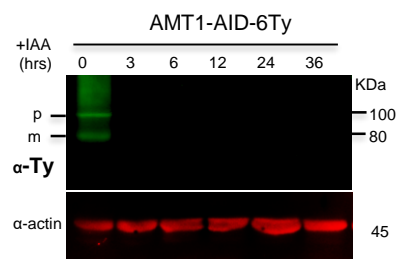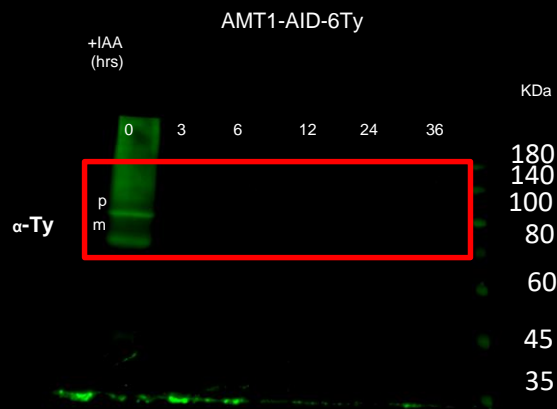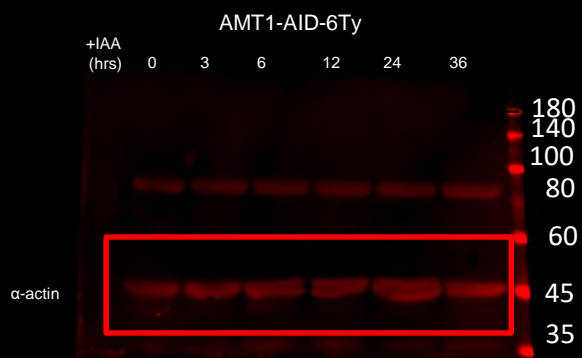

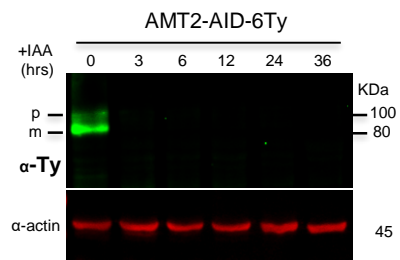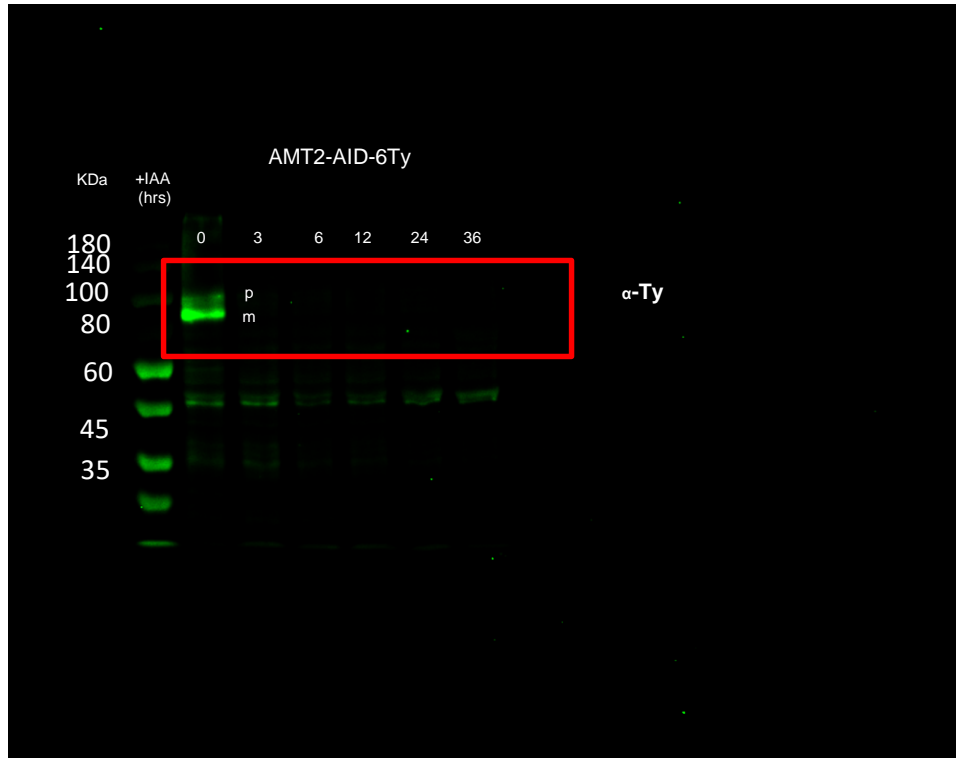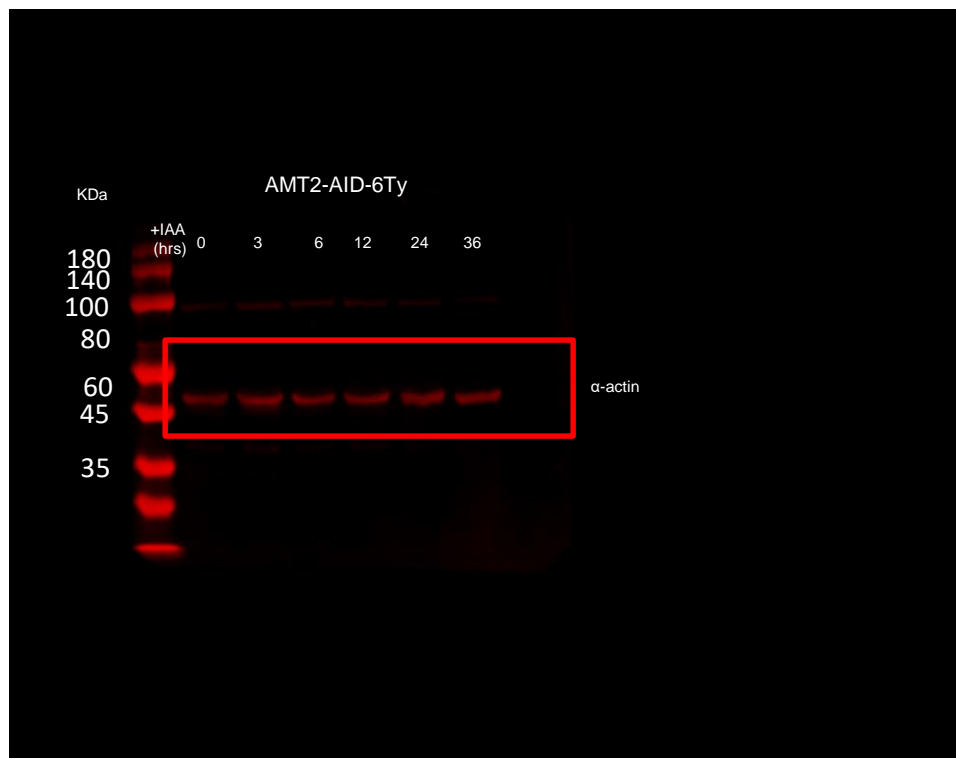

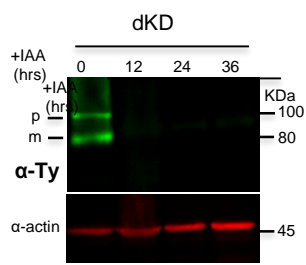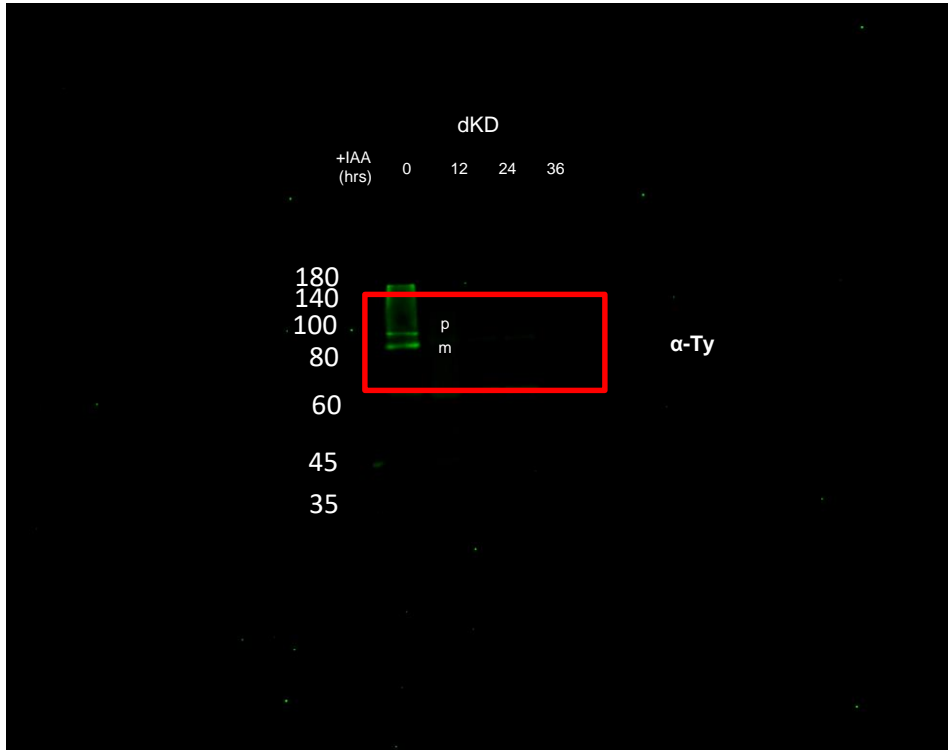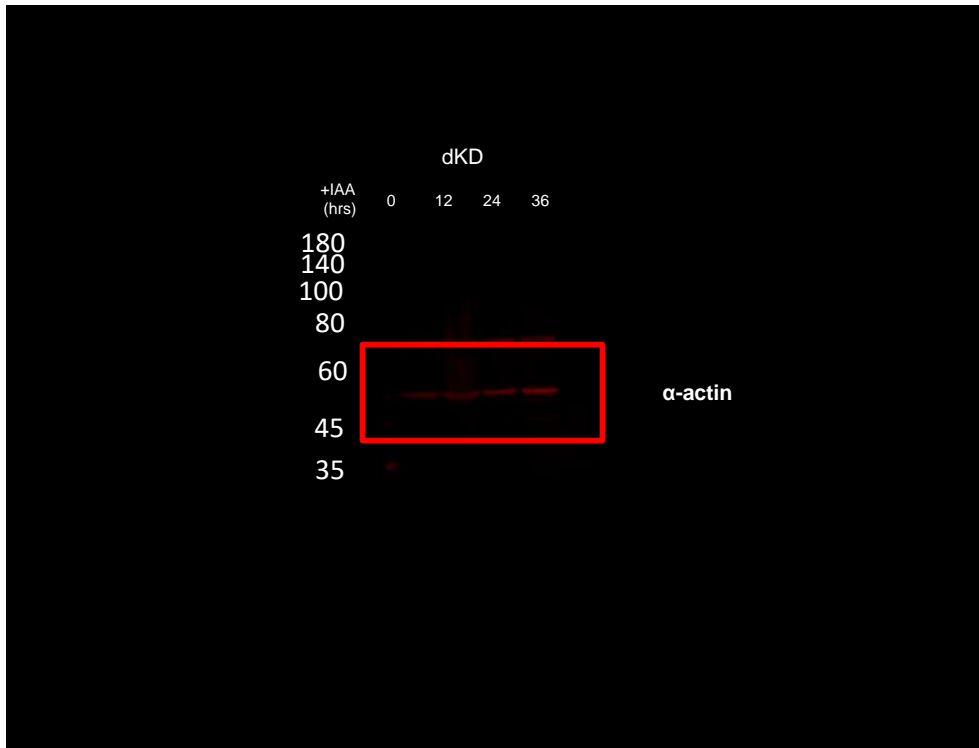

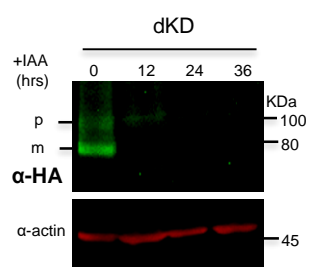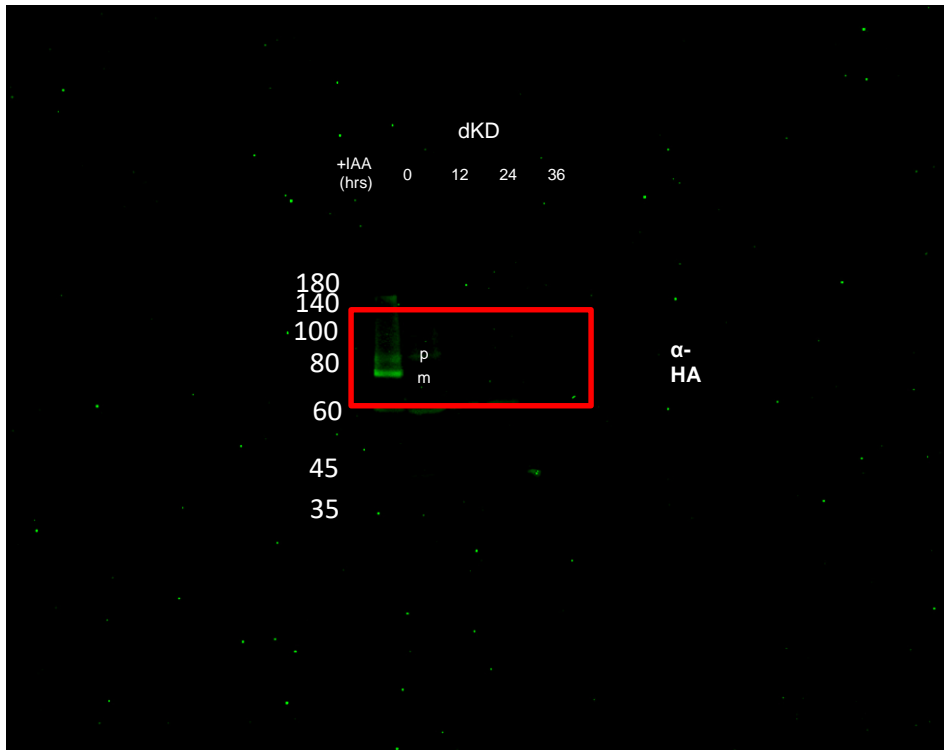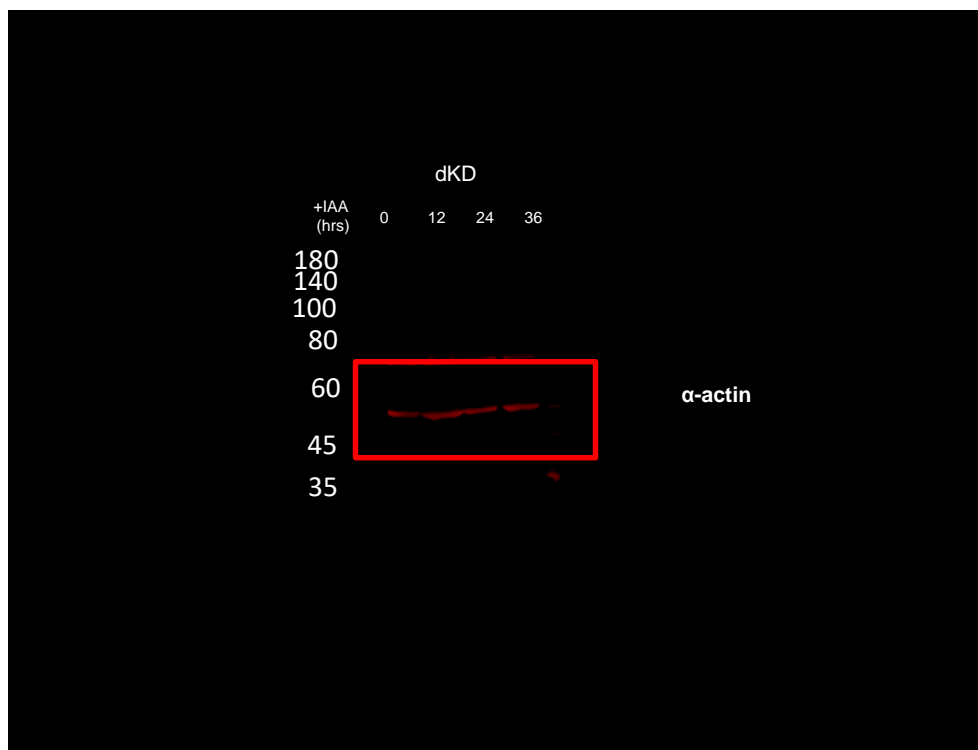

Supplement: Figure 3—source data 1. [file elife-88866-fig3-data1.zip › Figure 3-Source data 1.pdf]

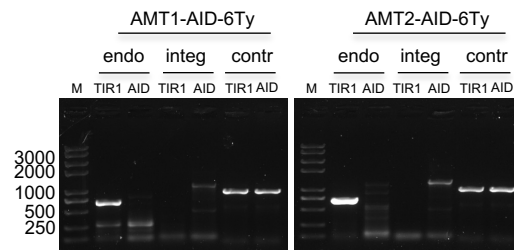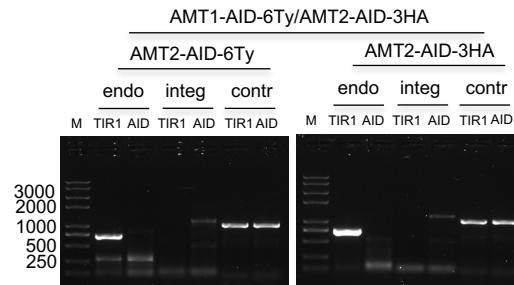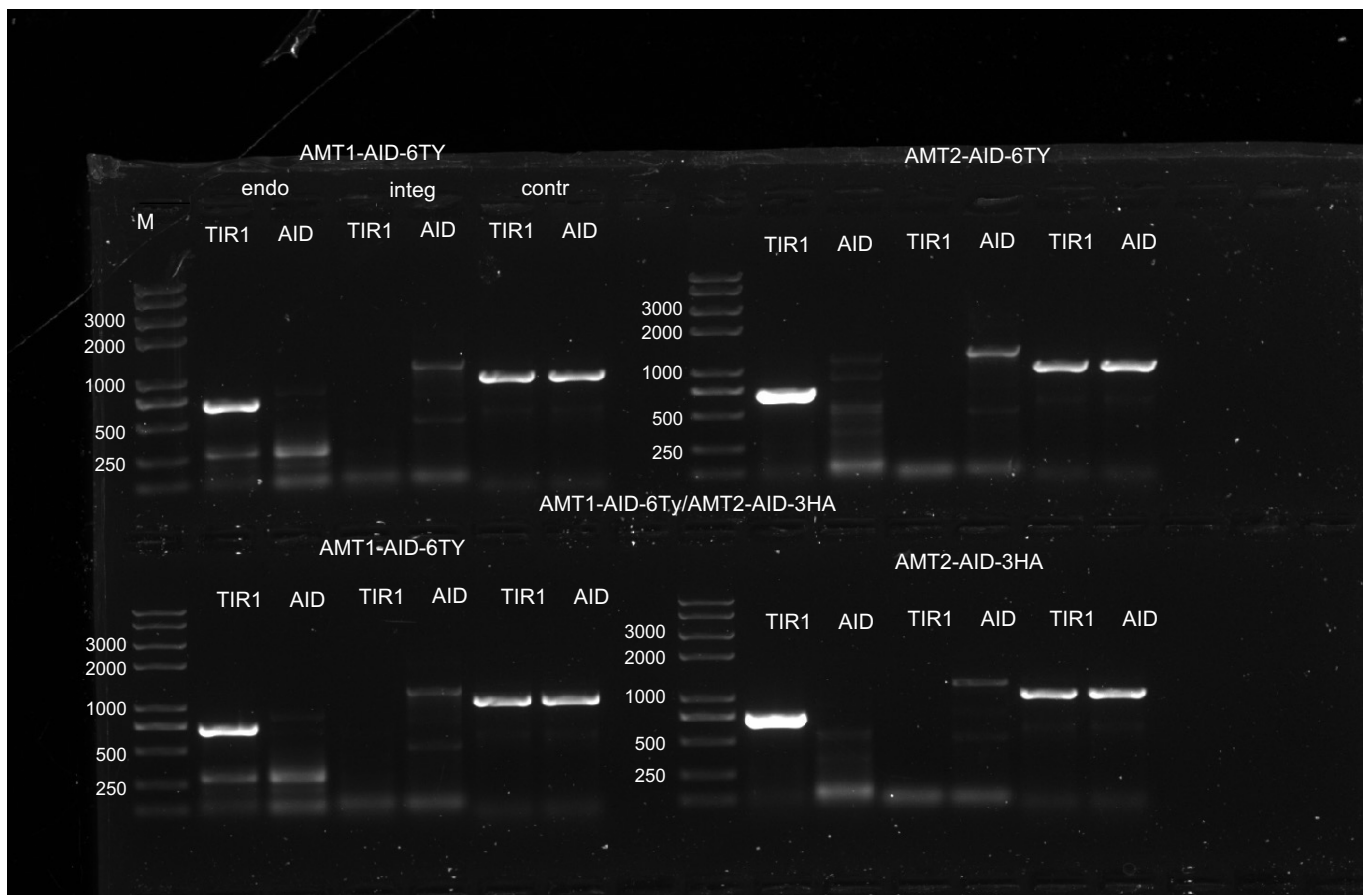

Supplement: Figure 3—figure supplement 2—source data 1. [file elife-88866-fig3-figsupp2-data1.zip › Figure3-figure supplement 2-Source data.pdf]

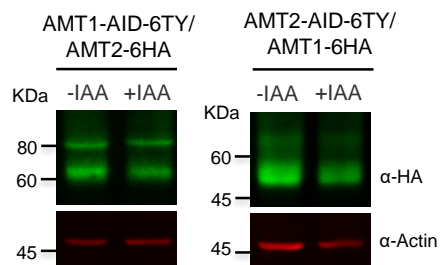

AMT1-AID-6TY/  
AMT2-6HA      AMT1-AID-6TY/  
AMT2-6HA

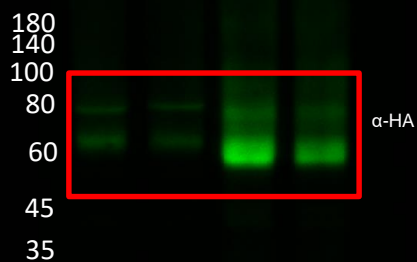

AMT1-AID-6TY/  
AMT2-6HA      AMT1-AID-6TY/  
AMT2-6HA

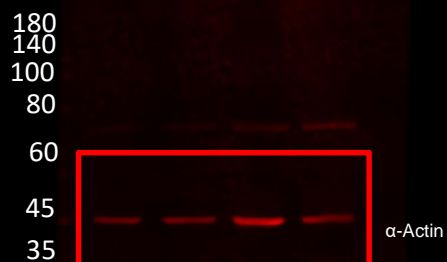

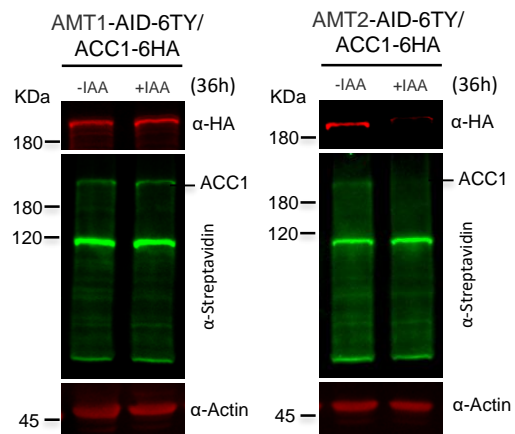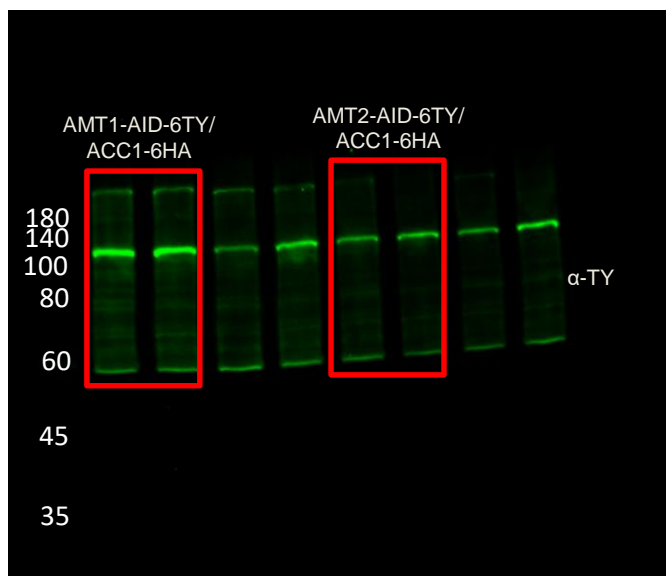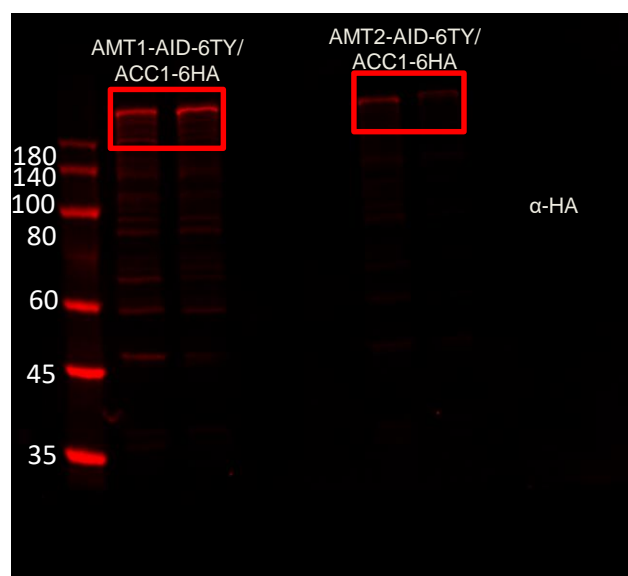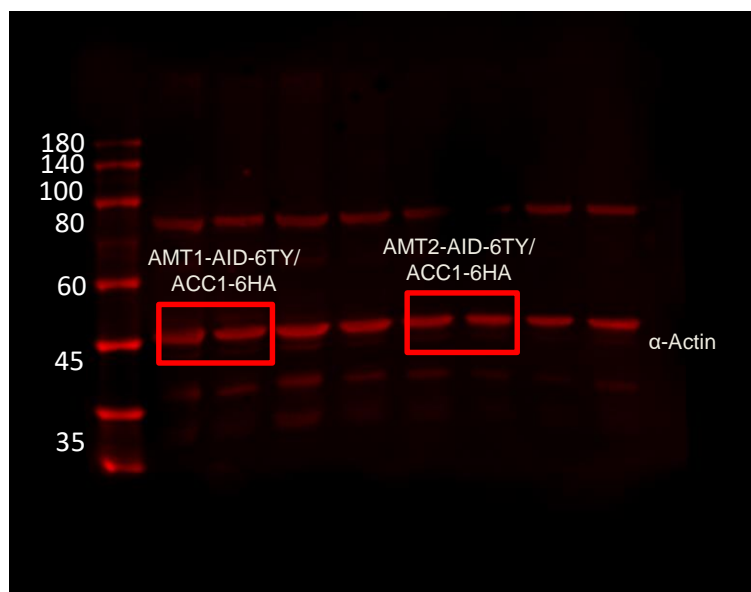

Supplement: Figure 5—source data 1. [file elife-88866-fig5-data1.zip › Figure 5-Source data.pdf]
